# Cost-effectiveness of larviciding for urban malaria control in Tanzania

**DOI:** 10.1186/1475-2875-13-477

**Published:** 2014-12-04

**Authors:** Mathieu Maheu-Giroux, Marcia C Castro

**Affiliations:** Department of Global Health & Population, Harvard School of Public Health, Boston, MA USA

**Keywords:** Malaria, Cost-effectiveness, Economic evaluation, Integrated vector management, Larval source management, Larviciding, *Bacillus sphaericus*, *Bacillus thuringiensis* var. *israelensis*

## Abstract

**Background:**

Larviciding for malaria control can contribute to an Integrated Vector Management (IVM) approach. This intervention is currently supported in settings where breeding habitats are ‘few, fixed, and findable’, such as urban areas of sub-Saharan Africa, but the knowledge base regarding the cost-effectiveness of larviciding is non-existent.

**Methods:**

Programme costs and effectiveness data were collected from the Dar es Salaam Urban Malaria Control Programme in Tanzania. Cost-effectiveness ratios (CER) were estimated from the provider and societal perspectives for standard indicators using different malaria transmission scenarios.

**Results:**

CER for microbial larviciding were highly dependent on the assumed baseline malaria incidence rates. Using the societal perspective, net CER were estimated (in 2012 US dollars) at $43 (95% uncertainty intervals [UI]: $15-181) per disability-adjusted life year averted (DALY) when malaria incidence was 902 infections per 1,000 individuals, increasing to $545 (95% UI: $337-1,558) per DALY at an incidence of 122 per 1,000. Larviciding was shown to be cost-effective in Tanzania for incidences as low as 40 infections per 1,000 people per year.

**Conclusion:**

This is believed to be the first study to estimate the cost-effectiveness of larviciding for urban malaria control in sub-Saharan Africa. The results support the use of larviciding as a cost-effective intervention in urban areas and managers of national malaria control programme should consider this intervention as part of an IVM approach.

**Electronic supplementary material:**

The online version of this article (doi:10.1186/1475-2875-13-477) contains supplementary material, which is available to authorized users.

## Background

Integrated Vector Management (IVM), as endorsed by the World Health Organization (WHO) [1], emphasizes rational decision-making, intersectoral collaboration, and the combination of different tools for vector control. Strategies included in an IVM approach should be based on local eco-epidemiological conditions with the aim of improving *‘the efficacy, cost effectiveness, ecological soundness and sustainability of interventions’*[2]. Currently, insecticide-treated nets (ITNs) and indoor residual spraying (IRS) are among the most widely used vector control methods in sub-Saharan Africa (SSA) [3]. The scaling-up of these two interventions during the last decade, coupled with improved access to early diagnosis and prompt treatment, has contributed to the important decline in malaria burden on this continent [4, 5]. Nevertheless, IRS and ITNs could be insufficient to achieve the long-term goal of malaria elimination in much of SSA [6, 7] and current gains might not be sustained ‘*without adapting to the changing threats to and opportunities for reducing transmission*’ [3].

An additional strategy for malaria control, Larval Source Management (LSM), the management of potential mosquito larval habitats, has had historical successes [8–12] and was one of the primary methods for malaria control until the 1950s, when IRS with DDT became the preferred control method [13]. Environmental management and larviciding could nevertheless play a role when other vector control interventions have achieved their maximum practical impact and/or in the malaria pre-elimination and elimination phases [13]. A recent systematic review of LSM interventions has shown that, under selected circumstances in various Asian and African settings, LSM can decrease malaria burden [14]. LSM should only be considered in specific contexts, however, as this type of intervention is likely to be most effective in areas where larval habitats are ‘*few, fixed, and findable*’ [15]. In SSA, these conditions are likely to be found in settings of focal and low to moderate transmission, such as urban environments, desert fringes, high altitudes, and some densely populated rural areas [13]. Further, LSM could contribute to IVM when dominant vectors are biting and/or resting outdoors (exophagic and exophilic behaviour) and to help manage insecticide resistance [13]. The knowledge base regarding the cost-effectiveness of LSM interventions is scarce, however. Environmental management was the subject of only one cost-effectiveness study that was based on the analysis of a colonial-era integrated malaria control programme carried out in copper mining communities of former Northern Rhodesia (present day Zambia) [16]. It is believed that only one cost analysis of larviciding programmes has been performed to date [17, 18] and the cost-effectiveness of this type of intervention remains to be assessed. This adds to the paucity of data on the cost-effectiveness of vector control interventions in urban areas.

The aim of this paper is thus to estimate the cost-effectiveness of larviciding for malaria control in urban areas of SSA, drawing from the recent large-scale community-based larviciding programme carried out by the *Urban Malaria Control Programme* (UMCP) in Dar es Salaam (United Republic of Tanzania) [19–24]. Cost-effectiveness ratios (CER) per malaria infection averted, malaria deaths prevented, and disability-adjusted life years (DALY) avoided are reported from both provider and societal perspectives for different transmission intensity scenarios and microbial larvicide formulations.

## Methods

### Dar es Salaam urban malaria control programme

This economic analysis is based on a large-scale larviciding intervention conducted in urban Dar es Salaam, Tanzania’s largest city and economic capital. The dominant malaria vectors are *Anopheles gambiae s.s.* and *Anopheles funestus.* These vectors transmit *Plasmodium falciparum* who is responsible for more than 90% of infections [25]. The principal types of breeding habitats encountered in Dar es Salaam are: drains, borrow pits, ponds, aquatic habitats associated with urban agriculture, and swamps [26]. Malaria transmission is year-round but exhibits seasonal variations related to the two rainy seasons: the short rains of October to December and the long rains of March to May.

The Urban Malaria Control Programme (UMCP) was launched in 2004 with the goal of developing a sustainable larviciding intervention as part of an integrated malaria strategy [21, 27, 28]. The UMCP targeted 15 urban wards, five in each of the three municipalities that composed Dar es Salaam (Temeke, Ilala, and Kinondoni), covering 56 km^2^ of the city and encompassing a population of 610,000 residents (2002 census) [29]. Larviciding was operationalized through a vertically managed community-based delivery system [17]. Routine mosquito surveillance and control was delegated to modestly paid community members called Community-Owned Resource Person (CORP) [21, 27, 28]. Larviciding was initiated in March of 2006 in three of the 15 UMCP wards (one in each municipality), and subsequently scaled-up to nine wards in May of 2007, and to the entire study region in April of 2008.

### Costing

Costing data for this study were extracted from the UMCP cost analysis described in Worrall [18] and Worrall and Fillinger [17]. Both studies adopted an ‘*ingredients approach*’ [30] to analyse costs, which is consistent with methods used for costing large-scale ITN and IRS programmes. The cost analysis was informed by data from the first phase of larviciding, when the intervention was operational in three wards, and mapping and larval surveillance activities were being carried out in the remaining wards [17, 18]; thus operational costs from these three wards were extrapolated for the entire study area. All resources used and the opportunity costs of existing inputs were taken into account. Specifically, the costs of the intervention include: community sensitization, training (including international consultants), field personnel, ward supervisors, larvicide purchase and distribution, transportation, materials, office space and furniture (including overheads), storage, and monitoring and evaluation (note that all research costs were excluded). Costs of capital items were spread over their estimated useful life and annualized using a 3% discount rate.

The UMCP used microbial larvicides for vector control manufactured by Valent BioSciences Corp. (Illinois, USA). The active ingredient of this product is a biological agent and is available in two formulations: 1) custom granule (CG) for hand application (*Bacillus sphaericus*; VectoLex®), and 2) water dispersible granule (WG) for liquid application (*Bacillus thuringiensis* var. *israelensis*; VectoBac®). Differences in international toxic units per milligrams of product between the two formulations result in higher costs for the CG formulation [17]. Although the UMCP made the programmatic decision to routinely apply CG, the impact on CERs of using the less expensive WG formulations will be explored.

For the purpose of this analysis, the larviciding intervention was presumed to be part of an ongoing programme and costs were, therefore, aggregated over 10 years (2004–2014) - 2004 being the pre-implementation phase when ward mapping, programme planning, and training occurred. Larviciding was operationalized starting in 2005. Other assumptions include that the intervention would not be scaled-up beyond UMCP wards and that the only increase in the number of persons protected would be due to population growth. To this end, ward-specific population counts from the 2002 and 2012 censuses were used and it was found that population growth averaged 1.62% over that period: from 610,000 in 2002 to 716,000 in 2012 [31, 32]. International technical consultants were assumed to be required for the first 5 years of the programme, time after which it was considered that capacity building, technical support, and troubleshooting of the intervention would no longer require international expertise. Results are reported based on the economic costs of the intervention as it is considered more appropriate for comparisons of interventions’ efficiency than using financial costs. All prices have been adjusted to 2012 US dollars (USD) using the US Gross Domestic Product (GDP) deflator [33] after being converted from local currency using average exchange rates for the year they were disbursed.

### Effectiveness data

The main clinical outcome reported by the UMCP is the prevalence of malaria infection, as determined by Giemsa-stained thick smear microscopy. Initial results from the first phase of the larval control intervention, restricted to children under five years of age (N = 4,450), suggested that the odds of malaria infection were decreased by 72% [27]. Further, anopheline larval abundance has been shown to be reduced by 96% during this same time period [21]. Analyses including individuals of all ages and using data from all phases of the larviciding intervention’s rollout (N = 64,537) have shown that the odds of malaria infection were 21% lower for individuals living in larviciding wards (Odds Ratio = 0.79; 95% Credible Intervals (CrI): 0.66-0.93) [28]. This logistic regression model was re-fitted in order to provide an effect size estimate on the relative risk scale. This yielded a prevalence ratio of 81% (95% CrI: 0.70-0.94). This effect size measure provides a conservative approximation of the rate ratio [34] and will be used to estimate the number of infections averted.

### Health outcomes

CERs are reported for the following health outcomes: malaria infections averted, malaria-associated deaths prevented, and disability-adjusted life years (DALY) avoided. One limitation of the UMCP data is that it collected information on prevalent cases, not incident ones. In order to estimate these three health outcomes, however, one needs a measure of incidence [35]. A two-component mixture of continuous-time Markov Chains was used to calculate incidence rates from UMCP data in these analyses (available from Castro *et al*. [36]).

Number of deaths prevented was estimated by multiplying the number of infections averted by the proportion of malaria cases found to be symptomatic and the case fatality rate. Of all prevalent malaria cases recorded by the UMCP, only 17% reported either having had a fever in the last two weeks before the survey or were found to have a body temperature higher than 37.5° Celsius at the time of the interview. Further, a clear relationship between age of prevalent malaria cases and occurrence of fever was not observed. For this reason, it was decided to assume that the proportion of new infections that would contribute malaria morbidity would remain constant across age groups. Malaria case fatality rate in the city of Dar es Salaam was available for 2006 from official Ministry of Health statistics. The reported case fatality rate of 0.63% (among symptomatic cases presenting at health facilities) is about a third of the average for mainland Tanzania (1.82%) [37].

DALYs were calculated by combining malaria morbidity and mortality. Years of life lost due to disability were obtained by multiplying the number of cases prevented by the condition’s disability weight and the average duration of that condition. The approach used by the 2010 update of the Global Burden of Disease (GBD) was adopted [38, 39] and, accordingly, age weighting and discounting of DALYs were not applied. Detailed description of the calculations can be found in Additional file 1.

### Provider’s resources savings

The provider’s perspective takes the viewpoint of the Tanzanian Ministry of Health and Social Welfare. Costs savings per malaria infection averted were estimated by taking into account 1) the proportion of symptomatic individuals that seek treatment at a health facility, 2) the proportion treated as outpatient, 3) the proportion diagnosed with microscopy, 4) the costs of diagnosing malaria using microscopy, 5) the cost of diagnosing malaria using a rapid diagnostic test (RDT), 6) the cost of treating an uncomplicated falciparum malaria with artemether-lumefantrine, 7) the cost of diagnostic and hospitalization of a complicated falciparum malaria case treated with intramuscular quinine dihydrochlorine, and 8) the proportion of symptomatic individuals seeking care through community health workers. Finally, any user fees for diagnosis and treatment that would be collected by health facilities were subtracted from costs savings.

After accounting for treatment-seeking behaviour, the provider’s cost of treating one symptomatic case of malaria was estimated to be of $5.15 (17% of malaria infections were assumed to be symptomatic). This latter amount was used to aggregate costs savings over the 10-year duration of the larviciding programme and to discount savings occurring in the future at a 3% rate. Detailed information on the cost function used can be found in Additional file 1.

### Society’s resources savings

It has been argued that the most relevant reference case in economic evaluations should reflect the societal perspective, where all costs and consequences of the intervention are aggregated without regards to whom they accrue [30, 40]. In Tanzania, it was estimated that 55% of all treatment costs of malaria in children under five years of age were borne by the household [41]. To estimate household costs in Dar es Salaam, the framework developed by Sicuri *et al.*[41] was generalized to individuals of all ages. Specifically, treatment-seeking behaviours, user fees, medicine costs, transportation costs, productivity losses due to clinical cases (or caring for sick children) of malaria, anemia, and neurological sequelae, and funeral costs were taken into account. The indirect costs per malaria infection (asymptomatic and symptomatic) and per death were estimated at $1.39 and $40.39, respectively (a detailed description of calculations can be found in Additional file 1). These costs were added to the provider’s costs savings to obtain the resources that would have been saved, from the societal perspective, for each infection averted.

The opportunity costs incurred by community members as a result of the larviciding intervention were not captured. Accounting for these costs would have negligible impact on the results of this economic evaluation, as it would only involve taking into account time to allow UMCP teams to access properties where breeding habitats could be found [17].

### Cost-effectiveness scenarios

A central feature of any cost-effectiveness analysis is the definition of the alternative to the studied intervention. WHO recommends a state of transmission without any intervention as the alternative [35]. This might not be the most realistic scenario as larviciding should be used as part of an IVM approach [1], in conjunction with other appropriate vector control measures [13]. To circumvent this issue and to enable generalization of these results, CERs were calculated as a function of incidence and the detailed results are presented considering three different scenarios:

- Scenario #1: Uses the baseline incidence for the year 2005, when malaria transmission was highest. The estimated incidence for that year was of 902 infections per 1,000 people per year that would result in 153 clinical malaria episodes per 1,000 people per year (assuming that 17% of cases will be symptomatic). For urban areas, malaria case incidence rates in this range have been described as characteristics of low transmission settings [42, 43].
- Scenario #2: This scenario assumes moderate malaria transmission that corresponds to what was observed in 2006 in the UMCP, when other control interventions were being scaled up. Malaria incidence has been estimated at 227 infections per 1,000 people per year.
- Scenario #3: The last scenario corresponds to the situation in which other malaria control interventions have already been scaled-up and achieved impact. This scenario assumes an annual malaria infection incidence of 122 infections per 1,000 people per year and is the lowest incidence recorded during the UMCP in 2008.

### Sensitivity analysis

One-way sensitivity analyses were used for parameters whose choice depends on methodological issues (e.g., larviciding formulation). Probabilistic analyses were performed to assess the impact of uncertainty in the effectiveness parameters, health outcomes, and costs. A Monte Carlo simulation model was built in R package v.2.15.1 [44] and parameters were re-sampled 100,000 times. The specific distributions from which the parameters were drawn are described in Additional file 2. The measure of dispersion reported for the CERs are the 95% uncertainty interval (UI) - the 2.5% and 97.5% percentiles of the Monte Carlo simulations. Finally, the uncertainty surrounding the cost-effectiveness of larviciding was summarized using cost-effectiveness acceptability curves.

### Ethical considerations

All UMCP data collection procedures were provided ethics approval from the Medical Research Coordination Committee of the National Institute for Medical Research, Ministry of Tanzania (Reference number NIMR/HQ/R.8a/Vol. IX/279 &234). Similarly, the Harvard School of Public Health’s Institutional Review Board also approved the research protocol (Protocol # 20323–101). Informed consent was obtained from all study participants or, on behalf of children under 18 years of ages, from their legal guardians.

## Results

The economic costs of the 10-year UMCP larviciding programme were evaluated at a present value of $5,111,234 (Table 1). The average economic cost per person protected per year (PPPY) was of $0.87. This number is lower than the economic cost PPPY year of $1.05 estimated by Worrall and Fillinger [17] because population growth was factored-in and international consultants were only included for the first five years of the programme.

**Table 1:** **Projected economic costs per year and population covered by the UMCP larviciding intervention**

Year	Economic costs*	Economic costs (Discounted)	Population covered
2004 (Y00)^†^	$147,882	$147,882	0
2005 (Y01)	$600,619	$583,125	637,406
2006 (Y02)	$600,619	$566,141	647,447
2007 (Y03)	$600,619	$549,652	657,880
2008 (Y04)	$600,619	$533,642	668,717
2009 (Y05)	$567,366	$489,415	679,973
2010 (Y06)	$567,366	$475,160	691,662
2011 (Y07)	$567,366	$461,320	703,797
2012 (Y08)	$567,366	$447,884	716,394
2013 (Y09)	$567,366	$434,839	729,469
2014 (Y10)	$567,366	$422,174	743,040
**Total**	$5,954,555	$5,111,234	6,875,784

Note: All prices are in 2012 US dollars.

*Economic costs for Y01-Y04 are higher because it was assumed that international consultants were required for capacity building, planning, and trouble-shooting of the intervention.

^†^Pre-implementation year (Y00) has a 6-month duration.

The first scenario assumed the highest urban malaria transmission and resulted in the most optimistic cost-effectiveness results with 1,178,999 malaria infections averted over the 10-year programme duration (Table 2). Larviciding would prevent 1,265 deaths and result in a total of 65,125 DALYs averted. It was evaluated that these cases would result in costs savings of $878,301 (at present value) for the provider (assuming that 17% of infections are symptomatic), and an additional $1,433,425 would be saved from the perspective of the society. The gross CER has been estimated at $4.3 per infection averted, $4,040 per death prevented, and $78 per DALY avoided. Taking into account costs savings, the cost of the programme per DALY avoided decreased to $65 from the provider’s perspective and to $43 from the societal perspective.

**Table 2:** **Number of cases averted, deaths prevented, disability-adjusted life years averted, and gross and net cost-effectiveness ratios for the larviciding intervention**

Scenarios	Total	Gross CER*	CER* Provider’s perspective	CER* Societal perspective
Scenario #1 - Incidence rate of 902 per 1,000				
Infection averted	1,178,999	$4.3	$3.6	$2.4
Death prevented	1,265	$4,040	$3,345	$2,213
DALY averted	65,125	$78.5	$65.0	$43.0
Scenario #2 - Incidence rate of 227 per 1,000				
Infection averted	296,228	$17.3	$16.5	$15.3
Death prevented	318	$16,077	$15,383	$14,250
DALY averted	16,363	$312.4	$298.9	$276.9
Scenario #3 – Incidence rate of 122 per 1,000				
Infection averted	159,282	$32.1	$31.3	$30.1
Death prevented	171	$29,900	$29,206	$28,073
DALY averted	8,798	$580.9	$567.4	$545.4

Note: All prices are in 2012 US dollars.

*CER: Cost-Effectiveness Ratio.

When considering the second transmission scenario, the number of infections averted was much smaller (Table 2). Consequently, the present value of costs saved by averting cases from the provider’s perspective was of $220,677 and gross and net CER from both the provider and societal perspectives were similar. The last scenario used a very low incidence rate and, as expected, the CER were the least cost-effective of all scenarios. It was estimated that larviciding would avert 159,282 malaria infections. Again, gross CER and net CER from the provider and societal perspective were of the same order of magnitude.

### One-way sensitivity analyses – Larviciding formulations

If a water dispersible formulation was used, the present value of the 10-year larviciding programme’s costs would be reduced to $4,076,908 (20% less than the costs associated with the custom granule formulation), and the average economic cost per person protected per year would be of $0.69. Assuming that the water dispersible formulation has the same efficacy as the custom granule used by the UMCP, the larviciding programme becomes more cost-effective (Table 3). In fact, net societal CERs ranged from $1 to $24 per malaria infection averted, and from $27 to $428 per DALY averted depending on the scenarios under considerations.

**Table 3:** **Impact on gross and net cost-effectiveness ratios of changing the formulation from custom granule to the less expensive water dispersible formulation**

Scenarios	Gross CER*	CER* Provider’s perspective	CER* Societal perspective
Scenario #1 - Incidence rate of 902 per 1,000			
Infection averted	$3.5	$2.7	$1.5
Death prevented	$3,222	$2,528	$1,395
DALY averted	$62.6	$49.1	$27.1
Scenario #2 - Incidence rate of 227 per 1,000			
Infection averted	$13.8	$13.0	$11.8
Death prevented	$12,824	$12,130	$10,997
DALY averted	$249.2	$235.7	$213.7
Scenario #3 - Incidence rate of 122 per 1,000			
Infection averted	$25.6	$24.9	$23.6
Death prevented	$23,850	$23,155	$22,023
DALY averted	$463.4	$449.9	$427.9

Note: All prices are in 2012 US dollars.

*CER: Cost-Effectiveness Ratio.

### Probabilistic sensitivity analysis

**Figure 1:**
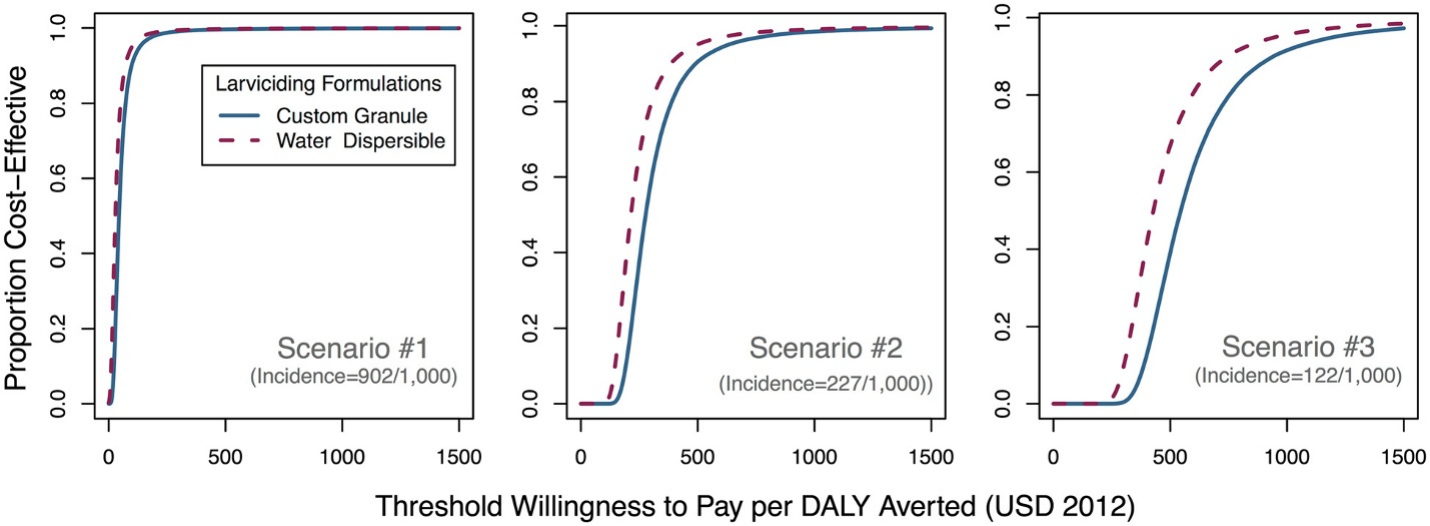
**Cost-effectiveness acceptability curves (societal perspective) for larviciding with the custom granule and water dispersible formulations under the three malaria transmission scenarios.**

**Figure 2:**
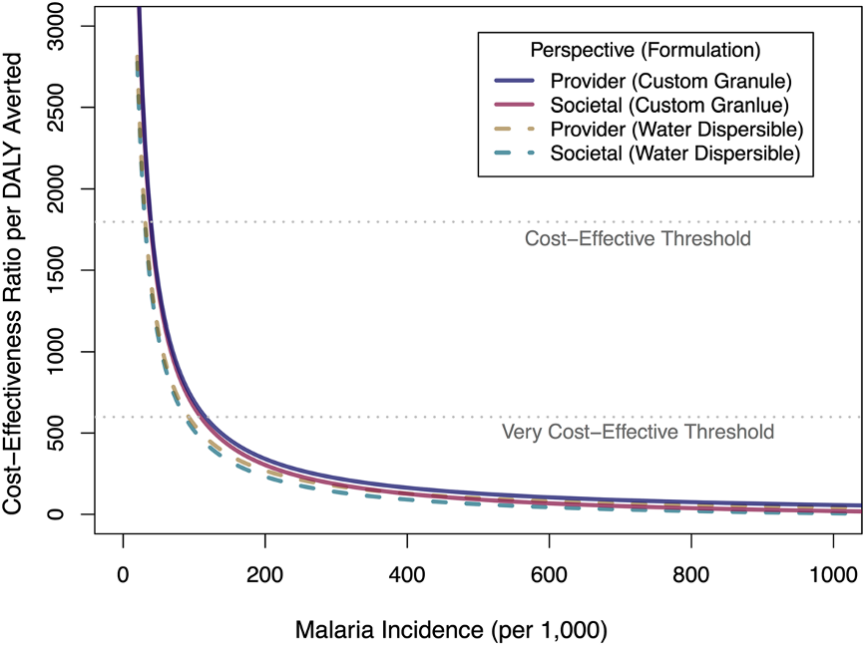
**Net cost-effectiveness of larviciding (custom granule formulation) per disability-adjusted life years as a function of malaria incidence for the provider and societal perspectives.** The very cost-effective threshold is defined as a cost-effectiveness ratio below the per capita Gross Domestic Product (GDP) of Tanzania ($599 USD), and the cost-effective threshold to three times the per capita GDP.

The uncertainty surrounding parameters’ estimates was explored through a probabilistic sensitivity analysis. CERs per infection averted, death prevented, and DALY avoided are presented with their 95% UI in Table 4. Uncertainty in parameters to calculate costs savings per malaria cases averted did not have an overwhelming influence on the CER, as demonstrated by the relatively high overlap between both gross and net CERs. Because of the relatively wide credible intervals around the prevalence ratios for the larviciding intervention (i.e., 95% CrI: 0.70-0.94) [28], there is about a 4-fold difference between the 2.5^th^ and 97.5^th^ percentiles of the simulated distributions for the gross CER. Net societal CER per additional DALY avoided had a 95% uncertainty interval of $15-181 for the scenario where transmission was highest, $165-822 for the second scenario and $337-1,548 for the lowest transmission scenario.Cost-effectiveness acceptability curves show the proportion of simulations that were cost-effective for a range of policy-makers’ willingness to pay (Figure 1). For the scenario where transmission was highest, 95% of Monte Carlo simulations had a willingness to pay threshold under $154 for an additional DALY averted (societal perspective and the custom granule formulation). This number increased to $652 and $1,225 for the second and third scenarios, respectively. To generalize these findings, net CERs were computed from the provider and societal perspectives for a range of incidence rates. Figure 2 shows that, except for very low incidences (i.e., <40 infections per 1,000 people per year), larviciding can be considered cost-effective.

**Table 4:** **Impact of probabilistic sensitivity analyses on gross and net cost-effectiveness ratios for the three malaria incidence scenarios**

Scenarios	Gross CER* [95% UI ^†^ ]	CER* Provider perspective [95% UI ^†^ ]	CER* Societal perspective [95% UI ^†^ ]
Scenario #1 - Incidence rate of 902 per 1,000			
Infection averted	[$3-12]	[$2-11]	[$1-10]
Death prevented	[$2,593-11,110]	[$1,879-10,399]	[$793-9,346]
DALY averted	[$50-215]	[$36-201]	[$15-181]
Scenario #2 - Incidence rate of 227 per 1,000			
Infection averted	[$11-47]	[$10-11]	[$9-46]
Death prevented	[$10,321-44,217]	[$9,612-43,503]	[$8,543-42,438]
DALY averted	[$200-856]	[$186-842]	[$165-822]
Scenario #3 - Incidence rate of 122 per 1,000			
Infection averted	[$21-88]	[$20-87]	[$19-86]
Death prevented	[$19,195-82,232]	[$18,491-81,503]	[$17,419-80,444]
DALY averted	[$372-1,592]	[$358-1,578]	[$337-1,558]

Note: All prices are in 2012 US dollars.

*CER: Cost-Effectiveness Ratio.

^†^95% UI: 95% Uncertainty Interval.

## Discussion

This paper presents results from the first economic evaluation of a large-scale larviciding intervention for malaria control under programmatic conditions. The cost-effectiveness of larviciding has been shown in this study to be highly dependent on the assumed baseline malaria incidence. WHO proposed that interventions with a CER per DALY averted less than a country’s per capita Gross Domestic Product (GDP) could be regarded as ‘*very cost-effective*’ and those for which the cost-effectiveness is less than three times the country’s per capita GDP as ‘*cost-effective*’ [35, 45]. Given Tanzania’s per capita GDP of $599 USD (2012), larviciding can be considered very cost-effective under a wide variety of transmission scenarios. Even low transmission settings with incidences above 40 infections per 1,000 people per year had CER that fell within the range of cost-effective interventions. With regards to the three malaria transmission scenarios, it was found that, even for the lowest malaria transmission scenario, 61% of Monte Carlo simulations fell below the very cost-effective threshold (societal perspective) and 98% of them below the cost-effective threshold.

These analyses also suggest that, if the same efficacy is assumed for both types of larviciding formulation, using a water dispersible larvicide is more cost-effective. Indeed, the provider CER for urban settings with the highest malaria transmission (Scenario #1) was estimated to be of $49 per DALY avoided (provider’s viewpoint), $27 if the societal perspective was adopted. In practice, the use of both formulations will likely be required as they are designed for different aquatic habitats: water dispersible granule being suited for open and non-vegetated breeding habitats, whereas the custom granule formulation is designed for habitats with emergent vegetation [17]. Hence, depending on the relative abundance of each type of aquatic habitats, the CER for larviciding should fall within the CERs calculated for the custom granule and water dispersible formulations.

Contextualizing these results is challenging because of inherent differences of cost-effectiveness studies of other malaria vector control interventions. A systematic review of economic evaluations of ITN and IRS programmes suggested that these interventions are highly cost-effective in rural areas with a median CER per additional DALY averted of $27 (range $8.15-110) and $143 (range $135-150) for ITN and IRS, respectively (in 2009 USD) [46]. Although this same review reported higher median financial costs per person protected per year for ITN of $2.20 and IRS of $6.70 (in 2009 USD) - as compared to $1.05 for the UMCP larviciding programme - the CERs estimated here are generally higher. A number of reasons can explain this differential and economic evaluations studies of ITN and IRS interventions conducted in SSA were systematically reviewed to address this point (see Additional file 3 for details on this systematic review).

First, protective efficacy for larviciding is lower than that of ITN and IRS [47, 48]. It was previously estimated that larviciding reduced the odds of malaria parasitaemia by 21% in the general population covered by UMCP activities [28]. The effectiveness estimate used in this economic evaluation can be considered conservative, however, since larviciding exhibited a greater protective effect for children under five years of age (i.e., Odds Ratio = 0.61; 95% Credible Interval: 0.46-0.80) [28]. Although the evidence based on the effectiveness of ITN is fairly robust [47], a recent Cochrane review of IRS interventions concluded that ‘*the number of high quality trials are too few to quantify the size of effect*’ [48]. Nevertheless, out of the seven IRS cost-effectiveness studies reviewed here, three of them assumed that the effect size of IRS was equal to that of ITN [49–51]. Thus, economic analyses of some of the IRS interventions could be considered imprecise.

Second, economic evaluation of ITN studies almost exclusively focus on the group of children at highest risk of malaria morbidity and mortality: children under five years of age. The ability to deliver ITN to the specific age group where malaria burden is highest decreases costs while maximizing health gains. Targeting larviciding, or to a lesser extent IRS, to children under five years of age is not in the realm of possibilities as these are population interventions. Finally, the baseline incidence rates used in this study comprised both asymptomatic and symptomatic infections whereas most other studies assumed that all cases would be symptomatic. In fact, the three malaria incidence scenarios entail that there would be between 21 (scenario #3) and 180 symptomatic cases (scenario #1) per 1,000 individuals per year. The median baseline malaria case incidence (symptomatic) used in the reviewed studies was of 900 and of 1,184 infections per year per 1,000 individuals for the ITN and IRS studies, respectively. Although these incidence rates fall into a realistic range for most endemic rural areas of SSA, malaria incidence is assumed to be much lower in urban areas. This last point is important because, although the costs of ITN and IRS programmes should remain relatively stable in urban areas, lower malaria incidence rates would reduce the number of DALY averted and increase CERs of these interventions. The same can be said of malaria case fatality rates that are generally lower in urban areas where prompt access to diagnostic and early treatment services are generally easier. This partly explains why the CER per death averted estimated for larviciding in urban Dar es Salaam is higher than the one reported for ITN and IRS in rural areas [46]. Importantly, the reviewed studies were almost exclusively conducted in rural areas. The question of which malaria control intervention is most cost-effective in urban settings, therefore, remains an open one.

Four potential methodological limitations of this study need to be acknowledged. The first concerns the generalizability of these results. This economic evaluation concerns a single larviciding programme in Dar es Salaam, where the costs were extrapolated from the first phase of larviciding, when only three out of the 15 wards where carrying-out the intervention [17]. If density of larval habitats in the other wards were higher or lower than that in these three wards, costs could have been under- or overestimated. Further, our results are likely to be generalizable only to other urban areas with similar malaria epidemiology. The estimated net CERs, that take into account costs savings from the provider and the societal perspectives, are unlikely to apply in settings where health systems characteristics, treatment seeking behaviors, and wages are drastically different. Second, health outcomes were not discounted in this economic evaluation, in accordance with the methodology adopted by the 2010 GBD update. Discounting DALYs at 3%, however, would have yield a net CER (society’s perspective) of $82, $528, and $1,040 per DALY averted for the first, second, and third transmission scenario, respectively. This increase of the CER by a factor of two highlights the impact of social value choices in economic evaluation. Yet, even when discounting health outcomes, larviciding remains below the cost-effective threshold for all scenarios (and below the very-cost effective threshold for scenarios #1 and #2). Third, using other protective measures such as ITN, window screening, and closed ceilings are believed to have synergistic effects with the larviciding intervention [28]. Not taking these synergies into account could underestimate the population impact of larviciding and the cost-effectiveness of the intervention. Finally, health insurance coverage, which could affect CER for both the provider and societal perspectives, was not taken into account. Given that health coverage is relatively low in Dar es Salaam, with only 7.8% of women and 5% of men aged 15–49 years of age having any form of insurance [52], this omission is, however, unlikely to dramatically impact the results.

In conclusion, this economic evaluation of the Dar es Salaam UMCP larviciding programme has shown that, according to commonly used GDP thresholds, this intervention is very cost-effective in most transmission settings where malaria incidence is above 110–116 infections per 1,000 per year (above 40 infections per 1,000 to be deemed cost effective). This study also lends support for the Tanzanian National Malaria Control Programme strategic plan to scale-up larviciding interventions by 2020 to selected urban areas of the country [53]. Given limited health budgets, however, decision-makers should still prioritize scaling-up ITN and IRS in rural areas because larviciding interventions have been shown to be more costly when the density of breeding habitats is high and/or the population density is low [17, 18]. Once coverage of these interventions is satisfactory in highly endemic areas, larviciding could be part of an IVM approach for malaria control, if local conditions warrant its use. This is especially true if other interventions have achieved their maximum impact and/or if the National Malaria Control Programme of a specific country wishes to move forward from malaria control to the pre-elimination and elimination phases. Finally, this study also highlights the lack of cost-effectiveness analyses for malaria control in urban areas of SSA, and it remains unknown which combinations of interventions (e.g., ITN, IRS, LSM) are most cost-effective in such settings.

## Electronic supplementary material

Additional file 1: **Details on the methodology and assumptions used in the cost-effectiveness analysis of larviciding for urban malaria control.** (PDF 339 KB)

Additional file 2: **Distributions and parameter values used for the probabilistic sensitivity analysis of cost-effectiveness of larviciding for urban malaria control.** (PDF 117 KB)

Additional file 3: **Systematic review of cost-effectiveness analyses of insecticide-treated bed nets and indoor residual spraying for malaria control in Africa.** (PDF 478 KB)

## Abbreviations

CER: Cost-effectiveness ratio
CORP: Community owned resource persons
DALY: Disability adjusted life year
GBD: Global Burden of Disease
GDP: Gross domestic product
ITN: Insecticide treated net
IRS: Indoor residual spraying
IVM: Integrated vector management
LSM: Larval source management
PPPY: Per person protected per year
SSA: Sub Saharan Africa
UI: Uncertainty interval
UMCP: Urban malaria control programme.
