# Supplementary material for: Cost-effectiveness of larviciding for urban malaria control in Tanzania
Source: Malar J. 2014 Dec 4;13:477. doi: 10.1186/1475-2875-13-477 (PMC4289051; doi:10.1186/1475-2875-13-477)
Supplement: Supplementary file 1 — Additional file 1: Details on the methodology and assumptions used in the cost-effectiveness analysis of larviciding for urban malaria control. (PDF 339 KB) [file 12936_2014_3657_MOESM1_ESM.pdf]

ADDITIONAL FILE 1

DETAILS ON THE METHODOLOGY AND ASSUMPTIONS USED IN THE COST-EFFECTIVENESS ANALYSIS OF LARVICIDING FOR URBAN MALARIA CONTROL

In this section, the methodology and assumptions used to estimate the cost-effectiveness ratios of larviciding for urban malaria control in Tanzania are described. This supplemental material is organized into three sections. First, the data and assumptions used to calculate the number of disability-adjusted life years averted (DALY) are described. Second, the methodology used to estimate provider's resources savings that would accrue by preventing malaria infections is presented. Third, the methodology adopted to estimate society's resources savings is defined.

Disability-adjusted life years

Previous Global Burden of Disease (GBD) assessments used the judgment of a small group of health-care professionals to assign disability weights to 483 sequelae of diseases and injuries. In contrast, the GBD 2010 update mapped 1,160 sequelae into 220 distinct health states, and weights were elicited through a large-scale multi-country respondent survey [1]. The health states and disability weights derived from this latest iteration of the GBD were used to estimate years of life lost due to disability. The seven malaria-related health states, proportion of cases assigned to each state, and their respective disability weights were abstracted from the GBD 2010 study report [2], and are presented in Table 1. The only exception is that the motor plus cognitive impairment state disease duration was estimated from the life expectancy at the average age of malaria death in Dar es Salaam (reliable information on the age distribution of neurological sequelae could not be found and the distribution of malaria deaths was used as the most plausible proxy).

Table 1 Description of health states, proportion of cases falling into each state, and disability weight used to calculate number of life years lost to disability.

Health State	Proportion of cases	Duration	Disability Weight
Mild case of acute infectious disease episode	66.3%	21 days	0.005
Moderate case of acute infectious disease episode	33.2%	21 days	0.053
Severe case of acute infectious disease episode	0.5%	21 days	0.210
Mild anemia	15.47%	28 days	0.005
Moderate anemia	20.28%	28 days	0.058
Severe anemia	4.61%	28 days	0.164
Moderate motor plus cognitive impairments	0.00906%	48.3 years	0.221

The proportion of malaria cases that would lead to mild, moderate, and severe anemia was calculated independently using local information. Following the approach outlined in the GBD 2010 update [3], the mean hemoglobin shift caused by malaria infections is estimated at 8.36 g/L and this shift was applied to the population distribution of hemoglobin levels in Tanzania. Separate distributions for individuals aged 0-4 years and 5-14 years, for men aged 15+ years, and for women aged 15+ years were used (Table 2). Information on hemoglobin distributions were obtained from the scientific literature for the city of Dar es Salaam for all age groups except for the 5-14 years old age group, which was based on data from coastal Tanzania. The hemoglobin shift was subtracted from the hemoglobin distributions described in Table 2,

and the increase in the prevalence of mild, moderate, and severe anemia was calculated using the appropriate age and sex-specific cut-off values for these anemia categories [3]. The average increase in prevalence across the different age and sex groups were combined using the distribution of malaria cases in these groups as weight (Table 3). The duration of malaria-attributable anemia was estimated to be the same for the three severity classes of anemia. Previous studies suggested that it usually takes 4-5 weeks to achieve hematological recovery following malaria infection [4-6] and a disability duration of 28 days was therefore used for these three anemia sequelae.

Table 2 Distribution of hemoglobin levels (g/L) used to calculate proportion of malaria cases that would lead to mild, moderate and severe anemia for different age and sex groups.

Population Group	Hemoglobin (g/L)		Reference
	Mean	SD	
Children aged 0-4 years (both sexes)	106.4	15.4	[7]
Children aged 5-14 years (both sexes)	111.5	13.9	[8]
Female aged ≥15 years	112.0	18.0	[9]
Male aged ≥15 years	128.0	16.0	[9]

Note: Mild anemia was defined as a hemoglobin level below 120 g/L for all age groups (except for males aged ≥15 years were a cut-off of 130 g/L was used). For moderate anemia, a threshold of 110 g/L was used (120 g/L for males aged ≥15 years). Severe anemia was defined using a cut-off hemoglobin level of 80 g/L (90 g/L for males aged ≥15 years).

Table 3 Age distribution of malaria cases and malaria deaths.

Age Group	Proportion of Malaria Cases*	Proportion of Malaria Deaths†
0-4 years old	19.1%	51.4%
5-14 years old	30.9%	10.9%
15-29 years old	26.9%	8.4%
30-44 years old	14.2%	11.9%
45-59 years old	5.7%	5.3%
60+ years old	3.3%	12.0%

*Age distribution of prevalent malaria cases estimated from the UMCP data.
†Age distribution of malaria deaths estimated from the Dar es Salaam Demographic Surveillance Site through verbal autopsies (including unspecified acute febrile illness).

Years of life lost were calculated by multiplying the expected number of deaths at each age by the remaining life expectancy at age of death in Tanzania [10]. The age distribution of malaria deaths (Table 3) was obtained from the Dar es Salaam Demographic Surveillance Site (DSS), conducted from 1994 to 2002 as part of the Adult Morbidity and Mortality Project (AMMP) [11], which, despite its name, collected information on individuals of all ages. Cause of death was ascertained through verbal autopsies. Because the cause of death was not specifically coded as malaria unless there was confirmatory evidence from another source (e.g. hospital records) [12], malaria deaths were considered to be those with a cause of '*malaria*' or '*unspecified acute febrile illness*', following the approach used by the AMMP and others [13, 14]. Social value choices, such as age weights, were not incorporated into DALYs and health

outcomes were not discounted, in accordance with the approach adopted in the GBD's 2010 update [15].

Provider's resource savings

A decision tree was developed to quantify the costs savings that would follow averting malaria cases in Dar es Salaam from the provider's perspective (Figure 1), which takes the viewpoint of the *Tanzanian Ministry of Health and Social Welfare*. Costs savings per malaria infection averted were estimated by taking into account 1) the proportion of symptomatic individuals that attended a health facility [P_{HF}], 2) the proportion treated as outpatient [P_{Out}], 3) the proportion diagnosed with microscopy [P_{Mic}], 4) the cost of diagnosing malaria using microscopy [CD_{Mic}], 5) the cost of diagnosing malaria using a rapid diagnostic test (RDT) [CD_{RDT}], 6) the cost of treating an uncomplicated falciparum malaria with artemether-lumefantrine (ALu) [CT_{Out}], 7) the cost of diagnostic and hospitalization of a complicated falciparum malaria case treated with intramuscular quinine dihydrochlorine [CDT_{In}], and 8) the proportion of symptomatic individuals seeking care through community health workers [P_{CHW}]. Finally, any user fees for diagnosis [UF_{Dx}] and treatment [UF_{Tx}] that would be collected by health facilities were subtracted from costs savings. Because children under five years of age are exempted from paying user fees, user fees were weighted by the probability of not having to pay them (using the age distribution of malaria cases described in Table 3). Costs savings per symptomatic malaria case averted were calculated using the formula below and parameter values are described in Table 4.

$$\text{Provider's Cost}_{Symptomatic} = P_{HF}\left\langle \left[P_{Out}\left\{(P_{Mic}*CD_{Mic})+(1-P_{Mic})*CD_{RDT})+CT_{Out}\right\}\right]+\left[(1-P_{Out})*CDT_{In}\right]-UF_{Dx}-UF_{Tx}\right\rangle$$
$$+P_{CHW}\left\langle CD_{RDT}+CT_{Out}-UF_{Dx}-UF_{Tx}\right\rangle$$

The proportion of symptomatic cases attending a health facility and seeking care through community health workers was estimated using UMCP data regarding the number of individuals who had a fever in the previous two weeks and sought advice or treatment at a health facility. That proportion was standardized using the age-distribution of prevalent malaria cases and it was estimated that, in Dar es Salaam, 65.7% of individuals infected with malaria (symptomatic) would seek treatment at a health facility and 4.4% through community health workers.

Once a malaria case present at the health facility, the proportion treated as outpatient and the number of hospitalizations need to be estimated. To this end, it was found from Tanzanian Ministry of Health data that 8.32% of all malaria cases presenting at health facilities were treated as in-patients [16]. The Tanzanian MoH's standard treatment guidelines states that '*where possible, laboratory investigations are mandatory*' [17]. Despite the fact that laboratory facilities are widely available in Dar es Salaam, a certain number of cases will be solely treated based on clinical symptoms (presumptive treatment). To produce consistent estimates of cost-effectiveness across interventions, all suspected malaria cases were assumed to be parasitologically confirmed either using RDT or microscopy, as per their National Malaria Control Program's guidelines. Specific data on the proportion of malaria diagnosis performed by RDT versus microscopy in Dar es Salaam could not be found. Instead, information from a study conducted in 2012 in two rural districts of Tanzania was used. This study reported that, among patients with fever who had a clinical diagnosis, RDT were used 56% of the time [18]. Provider's costs per diagnosis were informed by an economic evaluation conducted in six health facilities of Dar es Salaam that found that cost per diagnostic test (costs include laboratory materials and labor expenses) was $1.44 for RDT and $0.59 for microscopy (2008 USD) [19].

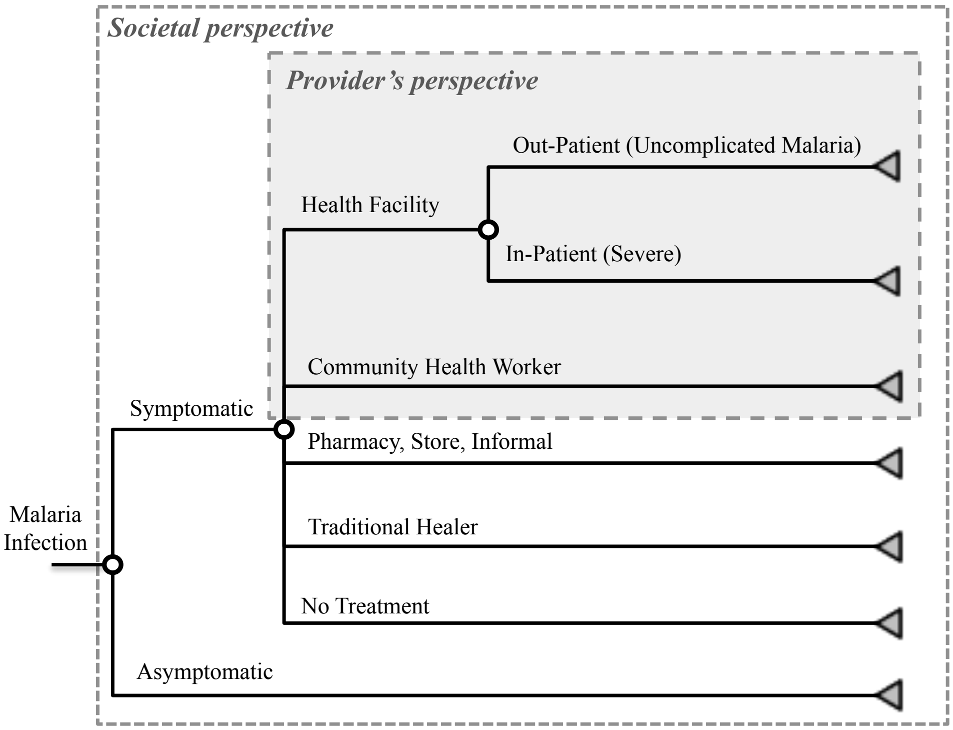

Figure 1 Decision tree model to calculate cost savings.

Table 4 Parameters and data sources used to calculate costs saved by averting one symptomatic malaria case.

Parameters	Value	Data Sources
P_{HF}	65.7%	UMCP data
P_{CHW}	4.4%	UMCP data
P_{Out}	91.7%	Ministry of Health data [16]
P_{Mic}	44%	Masanja *et al.* [18]
CD_{Mic}	$0.59	Harchut *et al.* [20] (1)
CD_{RDT}	$1.45	Harchut *et al.* [20] (1)
CT_{Out}	$1.85	Negotiated WHO/Coartem Price [21] (2)
CTD_{In}	$74.26	Lubell et al. [22] (3)
UF_{Dx}	$0.26	Ministry of Health data(4)
UF_{Tx}	$0.15	Ministry of Health data(4)

Note: All prices are in 2012 US dollars.
(1) These costs include overhead, labor costs, equipment, and general consumables.
(2) Drug price per tablet of 0.057 USD'09 with 20% adjustment for wastage, 10% for shipping, and 10% for CIF. A weighted average, using age as a proxy for weight, of the number of tablets required for an average ALu dose was calculated from the age distribution of cases in the UMCP data.
(3) Pooling data from all sites of this multi-center study. Costs include those for the antimalarial and other drugs, supportive treatment, diagnostic tests, treatment for adverse events and hotel costs for inpatient stay.
(4) The user fees are weighted by the probability that the patient is exempted from paying them (i.e., children under five years of age).

Standard treatment guidelines for Tanzania recommend ALu as first line treatment for uncomplicated malaria and quinine dihydrochlorine injection for complicated malaria [17]. All uncomplicated malaria cases were assumed to be treated as outpatients with ALu. To calculate cost per ALu treatment, the negotiated Novartis/WHO price of $0.057 per tablet was used (2009 USD) [21]. Because the number of tablets required per treatment is a function of a patient's weight, average treatment costs were calculated based on the weight distribution, proxied by age (as described in the MoH's Standard Treatment Guidelines [17]), of prevalent malaria cases in the UMCP data. Further, drug costs were inflated by 20% to adjust for wastage, an additional 10% for local transport, and 10% was added for international transport to properly reflect the costs incurred by the MoH [19]. All in-patients were presumed to have complicated malaria and estimated costs for treating such cases were abstracted from a recent multi-center trial of quinine versus parenteral artesunate for severe malaria [22]. The cost of treating a severe malaria case with quinine was estimated at $63.50 (2009 USD). This includes drugs, fluids, laboratories, and hotel costs – the latter being obtained from WHO's *choosing interventions that are cost-effective* framework – but excluded lifetime health care costs associated with neurological sequelae.

Individuals seeking care through community health workers were assumed to be diagnosed with RDT and treated with ALu. Because of lack of specific cost data on community health workers, the cost estimates of RDT from health facilities were used and the cost of ALu was presumed to remain the same. Using the cost function described above and accounting for treatment-seeking behavior, the provider's costs of treating one symptomatic case of malaria was estimated to be of $5.15 (17% of malaria infections are assumed to be symptomatic). The latter amount was used to aggregate costs savings over the 10-year duration of the larviciding program and to discount savings occurring in the future at a 3% rate.

Society's resources savings

To estimate household costs in Dar es Salaam, the framework developed by Sicuri *et al.* [23] was generalized to individuals of all ages (Figure 1). Specifically, treatment-seeking behaviors, fees, medicine costs, transportation costs, productivity losses due to clinical cases of malaria (or caring for sick children), anemia, and neurological sequelae, and funeral costs were taken into account.

Household direct costs are described in Table 5. Data from the UMCP was used to estimate the proportion of symptomatic malaria cases falling into five mutually exclusive treatment-seeking behaviors. For treatment in health facilities, user fees for diagnostic and treatment as well as transportation costs were taken into account. Because of the paucity of costs data regarding community health workers, societal costs were presumed to be the same as for those seeking treatment at health facilities, minus the transportation costs which is assumed to be null in the case of community health workers. For treatment in pharmacy/store, it was estimated that transportation costs would be negligible and that the only direct expenditure would be the cost of treatment with ALu. A small proportion of individuals sought care through traditional healers. Fees for such services were abstracted from the literature and it was premised that the same transportation costs reported by patients attending health facilities would apply for those reaching traditional healers. Individuals not seeking treatment were assumed to accrue no direct costs. Funeral costs were estimated from insurance premiums [24] and self-reported expenditure on funerals among individuals aged 15-59 years of age in Tanzania [25].

Table 5 Inputs and data sources to calculate household direct costs per symptomatic malaria episode.

	Household Direct Cost per Symptomatic Malaria Episode			
Treatment Seeking	Proportion(1)	Fee	Medicine Costs	Transportation Costs
Health Facility	65.70%	$0.26(2)	$0.15(4)	$0.29(6)
Community Health Worker	4.42%	$0.26(2)	$0.15(4)	-
Pharmacy/Store	3.95%	-	$0.77(5)	-
Traditional Healer	0.04%	$2.70(3)	-	$0.29(6)
No Treatment	25.90%	-	-	-

Note: All prices are in 2012 US dollars.
(1) UMCP data.
(2) Ministry of Health Data. User fee for diagnostic by community health worker is assumed to be equal to that of health facilities.
(3) Average between the fee reported by Sicuri et al. [23] and the one reported by Somi et al. [26].
(4) Based on the user fee for treatment in the health sector. User fee for treatment by community health worker is assumed to be equal to that of health facilities.
(5) Medicine costs for treating one malaria episode with artemether-lumefantrine. Cost estimate based on 798 private for-profit outlets in mainland Tanzania reported by Tougher et al. [27] and adjusted for the average weight (proxied by age) of malaria cases in Dar es Salaam.
(6) Average transportation cost of 259 patients from 6 health facilities of Dar es Salaam, as reported by Yukick et al. [19].

Household indirect costs were estimated by calculating productivity losses due to illness, anemia, and neurological sequelae. Changes in productivity were estimated using a human capital approach where market wage rates were used as a proxy for an individual's productive potential [28]. Time lost per symptomatic malaria episode has been estimated at 4.2 days in Tanzania [26]. Adult care-takers of sick children aged 0-9 years of age were presumed to also lose 4.2 days of productivity, and care-takers of children aged 10-14 to lose 1 day (25% of the time for younger children). Time lost in transportation or in medical facilities was not included to avoid double-counting, as affected individuals would already be out of economically productive activities due to malaria illness. The average monthly income in Dar es Salaam was abstracted from the 2006 Tanzanian Integrated Labour Force Survey (ILFS) database [29]. Taking into account the probability of unemployment, the average income in Dar es Salaam was calculated per 5-year age groups and the overall average income was weighted by the age distribution of malaria cases. Further, it was premised that care-takers of sick children would be women above 15 years of age so that such productivity losses would be calculated using the average income of this gender group – income for women in Dar es Salaam are roughly 60% lower than that of men. Note that caretakers of sick individuals or individuals affected by malaria may be non-economically active students that would also experience a reduction in their amount of earned education. Because of methodological difficulties in precisely quantifying the accumulation of human capital in this population, this type of indirect costs was not considered in the present economic evaluation.

Iron deficiency anemia can lead to important cognitive deficits in children and has negative impacts on adult work capacity [30]. Malaria is an important contributor of iron deficiency anemia, even in asymptomatic individuals [31, 32]. Productivity losses due to anemia are estimated to be of the order of 5% for blue-collar type work and can be as high as 17% for

heavy manual labor [30]. Malaria infections cause a mean hemoglobin decrease of 8.4 g/L [3, 33] and this shift was applied to the mean hemoglobin level of male and female aged 15 years of age. Distribution of hemoglobin levels for Dar es Salaam's adult population was abstracted from the literature (Table 2) and it was estimated that 16.9% of malaria cases would become anemic because of the infection. Productivity losses due to anemia were estimated by calculating average income per 5-year age groups, using data from Dar es Salaam in the 2006 ILFS [29], and assuming that income would be reduced by 5% due to anemia for the proportion of the population that became anemic as a result of malaria. Lost income was averaged using the age distribution of malaria cases as weights. The effect of malaria-attributable anemia on productivity was assumed to last for 4 weeks, as informed by studies on the duration of post-malaria hematological recovery [4-6].

Severe malaria has been associated with long-term cognitive impairments [34-37]. The impacts of such persistent neurological sequelae on lifetime productivity are clear but precise effect size estimates are unavailable. The proportion of malaria infections resulting in neurological sequelae has been estimated to be 0.00906% [2], and the productivity of individuals with such sequelae was assumed to be reduced by 15% - an estimate that can be considered conservative. Individuals were further presumed to be economically productive between the ages of 15 to 64 years and the average yearly earnings of this age group was calculated using data from Dar es Salaam, as reported in the 2006 ILFS [29]. Yearly earnings were estimated at $946 USD in this population so that the productivity losses due to neurological sequelae would be of $142 USD per year for affected individuals. The present value of lifetime productivity losses (LPL) due to neurological sequelae was estimated using the following formulae and a 3% discount rate:

$$LPL = \begin{cases} E\{[1-(1+r)^{-(e_x-15)}]/r\}*(1+r)^{-(15-Age_x)}; & \text{if } Age_x < 15 \text{ and } (e_x + Age_x) < 65 \\ E\{[1-(1+r)^{-(65-15)}]/r\}*(1+r)^{-(15-Age_x)}; & \text{if } Age_x < 15 \text{ and } (e_x + Age_x) \geq 65 \\ E\{[1-(1+r)^{-(e_x-Age_x)}]/r\}*(1+r)^{-(15-Age_x)}; & \text{if } Age_x \geq 15 \text{ and } (e_x + Age_x) < 65 \\ E\{[1-(1+r)^{-(65-Age_x)}]/r\}; & \text{if } Age_x \geq 15 \text{ and } (e_x + Age_x) \geq 65 \\ 0; & \text{if } Age_x \geq 65 \end{cases}$$

where E is the average yearly earnings, r is the discount rate (3%), e_x is the local life expectancy at age x, and Age_x is the age at which an individual develops neurological sequelae. The average lifetime productivity loss was then calculated using the age distribution of malaria deaths. Lifetime productivity loss due to premature mortality was not included in this analysis because of extreme uncertainty in estimates of an individual's lifetime consumption of goods/services (education, health, etc.) that would need to be deducted from lifetime earnings.

Household direct and indirect costs were then combined assuming that only 17% of new malaria infections would be symptomatic and that asymptomatic infections would not lead to any costs, except through anemia-attributable productivity losses. The following formulas were used to calculate the average societal cost per malaria infection and per malaria death:

$$\text{Societal Cost}_{Infection} = P_{Symp}\left\{\left[\sum_{i=1}^{5} \text{P}_{TSBi} * (Fee_i + MedCost_i + Transport_i)\right] + PL_{Ill}\right\} * + PL_{Hb} + (P_{NS} * LPL_{NS})$$

$$\text{Societal Cost}_{Death} = Cost_{Funeral}$$

where P_{Symp} is the proportion of infections that are symptomatic (17%); P_{TSBi} is the proportion of symptomatic individuals falling into each of the five treatment-seeking behaviors defined in Table 5; Fee_i, $MedCost_i$, and $Transport_i$ are the health fee, medicine costs, and transportation costs, respectively, incurred by individuals seeking care in each of these five categories (see Table 5); PL_{Ill} is the productivity loss due to being sick or caring for sick children (note that PL_{Ill} is a weighted average of productivity losses by age group and is equal to $5.25); PL_{Hb} is the productivity loss associated with anemia ($0.20); P_{NS} is the proportion of all malaria infection leading to neurological sequelae (0.009%); LPL_{NS} is the present value of lifetime productivity losses due to neurological sequelae ($2,263); and $Cost_{Funeral}$ is the cost of a funeral ($40.4).

REFERENCES

1. Salomon JA, Vos T, Hogan DR, Gagnon M, Naghavi M, Mokdad A, Begum N, Shah R, Karyana M, Kosen S, et al: **Common values in assessing health outcomes from disease and injury: disability weights measurement study for the Global Burden of Disease Study 2010.** *Lancet* 2012, **380:**2129-2143.

2. Lim S, Murray C, Rosenfeld L: **GBD 2010 Estimation Strategy Report for Malaria.** Seattle, WA: Institute for Health Metrics and Evaluation; 2012.

3. Jasrasaria R, Murray C, Naghavi M: **GBD 2010 Estimate Strategy Report for Anemia Envelope.** pp. 54: Institute for Health Metrics and Evaluation; 2012:54.

4. Price RN, Simpson JA, Nosten F, Luxemburger C, Hkirjaroen L, ter Kuile F, Chongsuphajaisiddhi T, White NJ: **Factors contributing to anemia after uncomplicated falciparum malaria.** *Am J Trop Med Hyg* 2001, **65:**614-622.

5. Doherty CP, Cox SE, Fulford AJ, Austin S, Hilmers DC, Abrams SA, Prentice AM: **Iron incorporation and post-malaria anaemia.** *PLoS One* 2008, **3:**e2133.

6. Sowunmi A, Gbotosho GO, Happi CT, Fateye BA: **Factors contributing to anaemia after uncomplicated Plasmodiumfalciparum malaria in children.** *Acta Trop* 2010, **113:**155-161.

7. NBS, ICF Macro: **Tanzania Demographic and Health Survey 2010.** pp. 451. Dar es Salaam, Tanzania: National Bureau of Statistics (NBS) [Tanzania] and ICF Macro; 2011:451.

8. Hall A, Bobrow E, Brooker S, Jukes M, Nokes K, Lambo J, Guyatt H, Bundy D, Adjei S, Wen ST, et al: **Anaemia in schoolchildren in eight countries in Africa and Asia.** *Public Health Nutr* 2001, **4:**749-756.

9. Kitange H, Swai A, Kilima P, Masuki G, Alberti K, McLarty D: **Anaemia is a major public health problem in Tanzania.** *Health Policy and Planning* 1993, **8:**413-424.

10. WHO: **World Health Statistics 2013.** pp. 168. Geneva, Switzerland: World Health Organization; 2013:168.

11. Mswia R, Whiting D, Kabadi G, Masanja H, Setel P: **Dar es Salaam DSS, Tanzania.** In *Population and Health in Developing Countries Volume 1 Population, Health, and Survival at INDEPTH Sites.* Edited by Network I. Ottawa, Canada: International Development Research Centre; 2002: 339

12. AMMP Team: **Policy implication of adult morbidity and mortality. End of phase one report.**: Adult Morbidity and Mortality Project. (United Kingdom Department for International Development and Government of the United Republic of Tanzania). 1997.

13. Adjuik M, Smith T, Clark S, Todd J, Garrib A, Kinfu Y, Kahn K, Mola M, Ashraf A, Masanja H, et al: **Cause-specific mortality rates in sub-Saharan Africa and Bangladesh.** *Bull World Health Organ* 2006, **84:**181-188.

14. Rowe A, Rowe S, Snow R, Korenromp E, Armstrong Schellenberg J, Stein C, Nahlen B, Bryce J, Black R, Steketee R: **Estimates of the burden of mortality directly attributable to malaria for children under 5 years of age in Africa for the year 2000 - Final Report.** Washington DC: Child Health Epidemiology Reference Group (CHERG); 2006.

15. Murray CJ, Vos T, Lozano R, Naghavi M, Flaxman AD, Michaud C, Ezzati M, Shibuya K, Salomon JA, Abdalla S, et al: **Disability-adjusted life years (DALYs) for 291 diseases and injuries in 21 regions, 1990-2010: a systematic analysis for the Global Burden of Disease Study 2010.** *Lancet* 2012, **380:**2197-2223.

16. MoH: **Annual Health Statistical Abstract Tanzania Mainland 2008.** (Welfare MoHaS ed. pp. 117. Dar es Salaam, United Republic of Tanzania2008:117.

17. MoH: **Standard Treatment Guidelines (STG) and the National Essential Medicines List (NEMLIT) for Mainland Tanzania - Third Edition.** (Welfare TURoTMoHaS ed. pp. 212. Dar es Salaam, United Republic of Tanzania2007:212.

18. Masanja IM, Selemani M, Amuri B, Kajungu D, Khatib R, Kachur SP, Skarbinski J: **Increased use of malaria rapid diagnostic tests improves targeting of anti-malarial treatment in rural Tanzania: implications for nationwide rollout of malaria rapid diagnostic tests.** *Malar J* 2012, **11:**221.

19. Yukich J, D'Acremont V, Kahama J, Swai N, Lengeler C: **Cost savings with rapid diagnostic tests for malaria in low-transmission areas: evidence from Dar es Salaam, Tanzania.** *Am J Trop Med Hyg* 2010, **83:**61-68.

20. Harchut K, Standley C, Dobson A, Klaassen B, Rambaud-Althaus C, Althaus F, Nowak K: **Over-diagnosis of malaria by microscopy in the Kilombero Valley, Southern Tanzania: an evaluation of the utility and cost-effectiveness of rapid diagnostic tests.** *Malar J* 2013, **12:**159.

21. WHO: **Global supply of artemether-lumefantrine before, during, and after the Memorandum of Understanding between WHO and Novartis.** pp. 6. Geneva, Switzerland: World Health Organization; 2011:6.

22. Lubell Y, Riewpaiboon A, Dondorp AM, von Seidlein L, Mokuolu OA, Nansumba M, Gesase S, Kent A, Mtove G, Olaosebikan R, et al: **Cost-effectiveness of parenteral artesunate for treating children with severe malaria in sub-Saharan Africa.** *Bull World Health Organ* 2011, **89:**504-512.

23. Sicuri E, Vieta A, Lindner L, Constenla D, Sauboin C: **The economic costs of malaria in children in three sub-Saharan countries: Ghana, Tanzania and Kenya.** *Malar J* 2013, **12:**307.

24. Dercon S, De Weerdt J, Bold T, Pankhurst A: **Group-based funeral insurance in Ethiopia and Tanzania.** *World Development* 2006, **34:**685-703.

25. Ngalula J, Urassa M, Mwaluko G, Isingo R, Ties Boerma J: **Health service use and household expenditure during terminal illness due to AIDS in rural Tanzania.** *Trop Med Int Health* 2002, **7:**873-877.

26. Somi MF, Butler JR, Vahid F, Njau JD, Kachur SP, Abdulla S: **Economic burden of malaria in rural Tanzania: variations by socioeconomic status and season.** *Trop Med Int Health* 2007, **12:**1139-1147.

27. Tougher S, Ye Y, Amuasi JH, Kourgueni IA, Thomson R, Goodman C, Mann AG, Ren R, Willey BA, Adegoke CA, et al: **Effect of the Affordable Medicines Facility--malaria (AMFm) on the availability, price, and market share of quality-assured artemisinin-based combination therapies in seven countries: a before-and-after analysis of outlet survey data.** *Lancet* 2012, **380:**1916-1926.

28. Neumann PJ: **Costing and perspective in published cost-effectiveness analysis.** *Med Care* 2009, **47:**S28-32.

29. NBS: **Analytical Report for the Integrated Labour Survey, 2006.** pp. 124. Dar es Salaam, Tanzania: National Bureau of Statistics; Ministry of Planning, Economy and Empowerment; Tanzania Gender Networking Programme; Minisry of Labour, Employment and Youth Development; 2007:124.

30. Horton S, Ross J: **The economics of iron deficiency.** *Food Policy* 2003, **28:**51-75.

31. Crookston BT, Alder SC, Boakye I, Merrill RM, Amuasi JH, Porucznik CA, Stanford JB, Dickerson TT, Dearden KA, Hale DC, et al: **Exploring the relationship between chronic undernutrition and asymptomatic malaria in Ghanaian children.** *Malar J* 2010, **9:**39.

32. Cercamondi CI, Egli IM, Ahouandjinou E, Dossa R, Zeder C, Salami L, Tjalsma H, Wiegerinck E, Tanno T, Hurrell RF, et al: **Afebrile Plasmodium falciparum parasitemia decreases absorption of fortification iron but does not affect systemic iron utilization: a double stable-isotope study in young Beninese women.** *Am J Clin Nutr* 2010, **92:**1385-1392.

33. Kassebaum NJ, Jasrasaria R, Naghavi M, Wulf SK, Johns N, Lozano R, Regan M, Weatherall D, Chou DP, Eisele TP, et al: **A systematic analysis of global anemia burden from 1990 to 2010.** *Blood* 2013.

34. Holding PA, Stevenson J, Peshu N, Marsh K: **Cognitive sequelae of severe malaria with impaired consciousness.** *Trans R Soc Trop Med Hyg* 1999, **93:**529-534.

35. John CC, Bangirana P, Byarugaba J, Opoka RO, Idro R, Jurek AM, Wu B, Boivin MJ: **Cerebral malaria in children is associated with long-term cognitive impairment.** *Pediatrics* 2008, **122:**e92-99.

36. Boivin MJ, Bangirana P, Byarugaba J, Opoka RO, Idro R, Jurek AM, John CC: **Cognitive impairment after cerebral malaria in children: a prospective study.** *Pediatrics* 2007, **119:**e360-366.

37. Carter JA, Mung'ala-Odera V, Neville BG, Murira G, Mturi N, Musumba C, Newton CR: **Persistent neurocognitive impairments associated with severe falciparum malaria in Kenyan children.** *J Neurol Neurosurg Psychiatry* 2005, **76:**476-481.
