# Supplementary material for: Cost-effectiveness of larviciding for urban malaria control in Tanzania
Source: Malar J. 2014 Dec 4;13:477. doi: 10.1186/1475-2875-13-477 (PMC4289051; doi:10.1186/1475-2875-13-477)
Supplement: Supplementary file 2 — Additional file 2: Distributions and parameter values used for the probabilistic sensitivity analysis of cost-effectiveness of larviciding for urban malaria control. (PDF 117 KB) [file 12936_2014_3657_MOESM2_ESM.pdf]

ADDITIONAL FILE 2

Table 1 Distributions and parameter values used for the probabilistic sensitivity analysis.

Parameters	Distribution*	Notes
EFFECTIVENESS		
Prevalence ratio for the intervention (log scale)	*Truncated Normal* (ln(0.81); 0.072)	1
DISABILITY-ADJUSTED LIFE YEARS		
Disability weight for infectious disease: mild	*Triangular* (min=0.002; max=0.011)	2
Disability weight for infectious disease: moderate	*Triangular* (min=0.033; max=0.081)	2
Disability weight for infectious disease: severe	*Triangular* (min=0.139; max=0.298)	2
Disability weight for anemia: mild	*Triangular* (min=0.002; max=0.011)	2
Disability weight for anemia: moderate	*Triangular* (min=0.038; max=0.086)	2
Disability weight for anemia: severe	*Triangular* (min=0.112; max=0.228)	2
Disability weight for cognitive impairments	*Triangular* (min=0.141; max=0.314)	2
Duration of a malaria episode (days)	*Uniform* (min=14, max=28)	3
Duration of anemia (days)	*Uniform* (min=21, max=35)	4
TREATMENT-SEEKING BEHAVIORS		
Proportion seeking care at/through health facility (PS_{HF}), community-health workers (PS_{TSB1}), pharmacy/store (PS_{TSB2}), traditional healers (PS_{TSB3}), and not seeking treatment (PS_{TSB4}).	PS_{HF} =*Triangular* (min=54%; max=81%)<br>$PS_{TSBi} = P_{TSBi}+[P_{TSBi}*(P_{HF}-PS_{HF})/(1-P_{HF})]$	5
COSTS SAVINGS (PROVIDER)		
Proportion treated as out-patient (P_{Out})	*Triangular* (min=72%; max=95%)	6
Proportion diagnosed using microscopy (P_{Mic})	*Triangular* (min=33%; max=55%)	7
Costs of diagnostic with microscopy (CD_{Mic})	*Triangular* (min=0.45$; max=0.75$)	7
Cost of diagnostic with RDT (CD_{RDT})	*Triangular* (min=0.52$; max=4.94$)	8
Outpatient's cost of treatment (CT_{Out})	*Triangular* (min=1.26$; max=5.22$)	9
Inpatient's cost of diagnostic and treatment (CTD_{In})	*Triangular* (min=58.38$; max=90.51$)	10
COSTS SAVINGS (SOCIETY)		
Fee for traditional healer	*Triangular* (min=0.21; max=5.19)	11
Cost of artemether-lumefantrine at pharmacy/store	*Triangular* (min=0.51; max=1.03)	12
Transportation costs to health facility/healer	*Triangular* (min=0.20; max=0.42)	13
Number of days lost to malaria episode (N) and to care for children aged 10-14 years of age (N_{10-14})	*N = Triangular* (min=2; max=5)<br>N_{10-14}=N*Uniform* (min=20%; max=30%)	14
Proportion of earnings lost to anemia	*Uniform* (min=2.5%; max=7.4%)	15
Proportion of earnings lost to neurological sequelae	*Triangular* (min=10%; max=20%)	16
Funeral costs	*Triangular* (min=30.53$; max=50.26$)	17

Note: All prices are in 2012 US dollars.
*The modes of the triangular distributions correspond to the values used to calculate the cost-effectiveness ratios.

Notes:

1- Adapted from Maheu-Giroux and Castro [1]. Because it is biologically impossible for larviciding to cause an increase in malaria incidence, the normal distribution was truncated for values above the null.

2- The 95% confidence intervals of the disability weights reported in the 2010 GBD study were used as minimum and maximum values of the triangular distribution [2].

3- For the duration of malaria episodes, the same distribution as the one reported in the GBD 2010 study was used [3].

4- The post-malaria recovery period reported in the literature [4-6] was used and ±1 week was added to that quantity.

5- Lowest and highest age-specific proportions of individual attending health facilities in the UMCP data. The other 4 treatment-seeking behaviors were rescaled such that the sum of these proportions would still be equal to 1. PS_{TSBi} refers to the re-sampled proportion whereas P_{TSBi} refers to the values described in Table 5.

6- Minimum for severe malaria is reported in Reyburn *et al*. [7]. An upper bound of 95% for this distribution was assumed.

7- Minimum and maximum values of the triangular distributions were calculated as ±25% the mode of the distribution.

8- Minimum is obtained by considering only the price of test (excluding labor), as reported by Harchut *et al*. [8]. Maximum value is taken from White *et al*. [9].

9- Minimum value is the negotiated WHO/Novartis price, excluding adjustments for wastage and transport. Maximum value corresponds to the price reported in the AFRO essential drug price indicator.

10- Minimum and maximum values as reported by Lubell *et al*. [10].

11- Minimum is reported by Somi *et al*. [11] and maximum by Sicuri *et al.* [12].

12- Minimum and maximum values correspond to the interquartile range reported by Tougher *et al*. [13].

13- Minimum and maximum values correspond to the 95% confidence interval reported by Yukich *et al*. [14].

14- Values for this triangular distribution are taken from the review of Chima *et al*. [15]. For children aged 10-14 years of aged, it was assumed that it would correspond to 20-30% of an adult illness time.

15- Minimum value taken by reducing by 50% the estimate of 5% loss in earning. Maximum value corresponds to a situation where 20% of workers in Dar es Salaam are involved in manual work where lost in earnings is estimated at 17% [16].

16- A uniform distribution was used to acknowledge the fact that prior information on this parameter is uncertain. The minimum and maximum values correspond to the authors' evaluation of a plausible conservative range.

17- Minimum value reported by Ngalula *et al*. [17] and maximum value by Dercon *et al*. [18].

References

1. Maheu-Giroux M, Castro M: **Impact of community-based larviciding on the prevalence of malaria infection in Dar es Salaam, Tanzania.** *PLOS One* 2013, **8:**e71638.

2. Salomon JA, Vos T, Hogan DR, Gagnon M, Naghavi M, Mokdad A, Begum N, Shah R, Karyana M, Kosen S, et al: **Common values in assessing health outcomes from disease and injury: disability weights measurement study for the Global Burden of Disease Study 2010.** *Lancet* 2012, **380:**2129-2143.

3. Lim S, Murray C, Rosenfeld L: **GBD 2010 Estimation Strategy Report for Malaria. (Unpublished Document; Personal Communication 2013).** pp. 305: Institute for Health Metrics and Evaluation; 2012:305.

4. Price RN, Simpson JA, Nosten F, Luxemburger C, Hkirjaroen L, ter Kuile F, Chongsuphajaisiddhi T, White NJ: **Factors contributing to anemia after uncomplicated falciparum malaria.** *Am J Trop Med Hyg* 2001, **65:**614-622.

5. Doherty CP, Cox SE, Fulford AJ, Austin S, Hilmers DC, Abrams SA, Prentice AM: **Iron incorporation and post-malaria anaemia.** *PLoS One* 2008, **3:**e2133.

6. Sowunmi A, Gbotosho GO, Happi CT, Fateye BA: **Factors contributing to anaemia after uncomplicated Plasmodiumfalciparum malaria in children.** *Acta Trop* 2010, **113:**155-161.

7. Reyburn H, Mbatia R, Drakeley C, Bruce J, Carneiro I, Olomi R, Cox J, Nkya WM, Lemnge M, Greenwood BM, Riley EM: **Association of transmission intensity and age with clinical manifestations and case fatality of severe Plasmodium falciparum malaria.** *JAMA* 2005, **293:**1461-1470.

8. Harchut K, Standley C, Dobson A, Klaassen B, Rambaud-Althaus C, Althaus F, Nowak K: **Over-diagnosis of malaria by microscopy in the Kilombero Valley, Southern Tanzania: an evaluation of the utility and cost-effectiveness of rapid diagnostic tests.** *Malar J* 2013, **12:**159.

9. White MT, Conteh L, Cibulskis R, Ghani AC: **Costs and cost-effectiveness of malaria control interventions--a systematic review.** *Malar J* 2011, **10:**337.

10. Lubell Y, Riewpaiboon A, Dondorp AM, von Seidlein L, Mokuolu OA, Nansumba M, Gesase S, Kent A, Mtove G, Olaosebikan R, et al: **Cost-effectiveness of parenteral artesunate for treating children with severe malaria in sub-Saharan Africa.** *Bull World Health Organ* 2011, **89:**504-512.

11. Somi MF, Butler JR, Vahid F, Njau JD, Kachur SP, Abdulla S: **Economic burden of malaria in rural Tanzania: variations by socioeconomic status and season.** *Trop Med Int Health* 2007, **12:**1139-1147.

12. Sicuri E, Vieta A, Lindner L, Constenla D, Sauboin C: **The economic costs of malaria in children in three sub-Saharan countries: Ghana, Tanzania and Kenya.** *Malar J* 2013, **12:**307.

13. Tougher S, Ye Y, Amuasi JH, Kourgueni IA, Thomson R, Goodman C, Mann AG, Ren R, Willey BA, Adegoke CA, et al: **Effect of the Affordable Medicines Facility--malaria (AMFm) on the availability, price, and market share of quality-assured artemisinin-based combination therapies in seven countries: a before-and-after analysis of outlet survey data.** *Lancet* 2012, **380:**1916-1926.

14. Yukich J, D'Acremont V, Kahama J, Swai N, Lengeler C: **Cost savings with rapid diagnostic tests for malaria in low-transmission areas: evidence from Dar es Salaam, Tanzania.** *Am J Trop Med Hyg* 2010, **83:**61-68.

15. Chima RI, Goodman CA, Mills A: **The economic impact of malaria in Africa: a critical review of the evidence.** *Health Policy* 2003, **63:**17-36.

16. Horton S, Ross J: **The economics of iron deficiency.** *Food Policy* 2003, **28:**51-75.

17. Ngalula J, Urassa M, Mwaluko G, Isingo R, Ties Boerma J: **Health service use and household expenditure during terminal illness due to AIDS in rural Tanzania.** *Trop Med Int Health* 2002, **7:**873-877.

18. Dercon S, De Weerdt J, Bold T, Pankhurst A: **Group-based funeral insurance in Ethiopia and Tanzania.** *World Development* 2006, **34:**685-703.
