# Supplementary material for: Cost-effectiveness of larviciding for urban malaria control in Tanzania
Source: Malar J. 2014 Dec 4;13:477. doi: 10.1186/1475-2875-13-477 (PMC4289051; doi:10.1186/1475-2875-13-477)
Supplement: Supplementary file 3 — Additional file 3: Systematic review of cost-effectiveness analyses of insecticide-treated bed nets and indoor residual spraying for malaria control in Africa. (PDF 478 KB) [file 12936_2014_3657_MOESM3_ESM.pdf]

ADDITIONAL FILE 3

SYSTEMATIC REVIEW OF COST-EFFECTIVENESS ANALYSES OF INSECTICE-TREATED BEDNETS AND INDOOR RESIDUAL SPRAYING FOR MALARIA CONTROL IN AFRICA

In order to contextualize the results of this economic evaluation of larviciding for malaria control, cost-effectiveness analyses of indoor residual spraying (IRS) and insecticide-treated bendets (ITN) for malaria control in SSA were systematically reviewed. This review will help examine the methodological issues and assumptions upon which these economic evaluations are based.

Methodology

PubMed was queried using the terms described below (Table 1). No time restriction was imposed. The search retrieved 402 publications that were evaluated based on either title alone (n=253) or title and abstract (n=149). The eligibility and exclusion criteria are described in Table 2. A total of 12 articles for IRS and 24 for ITN were selected based on their title and abstract but 5 IRS and 6 ITN studies were excluded after reading the paper (Table 3). Finally, one IRS study was identified through other means and included in the review. Hence, a total of 7 IRS and 18 ITN cost-effectiveness studies were included in the final review.

Results

A summary of the reviewed cost-effectiveness studies of IRS is present in Table 4. Cost-effectiveness studies of ITN is presented in Table 5.

Table 1 PubMed search query information for the systematic review

**PubMed Search:**<br>(malaria OR falciparum) AND (cost OR cost-effectiveness) AND ((ITN OR bed net* OR bednet* OR net OR long-lasting insecticide-treated net* OR LLIN) OR ((insecticide OR residual house spraying OR indoor residual spraying OR IRS))) AND (Africa)

Table 2 Eligibility and exclusion criteria for the systematic review

**Eligibility Criteria**	-Study conducted in Africa.<br>-Study reports cost-effectiveness of IRS or ITN.<br>-Malaria cases, malaria deaths, or DALYs are the health outcomes.
**Exclusion Criteria**	-Study not relevant to malaria.<br>-Literature review.<br>-Letter to the Editor.<br>-Studies conducted on packages of interventions where we cannot distinguish between IRS, ITN, or any combinations of interventions.<br>-Study is published in a language other than English, French, or Portuguese.

Table 3 Studies retrieved, selected, and included in the systematic review

Articles Retrieved (IRS and ITN combined)	402
Articles selected based on title and abstract	IRS = 12<br>ITN = 24
Final number of articles included in the review	IRS = 7<br>ITN = 18

Table 4 Summary of cost-effectiveness analyses of indoor residual spraying interventions conducted in sub-Saharan Africa.

Country	Year of Program	Currency	Age Group	Perspective	Cost PPY	Effect Size	Incidence per 1,000	CER / Infection	CER / Death	CER / DALY	Ref
Low and Middle Income Countries	NA	USD 2001	NA	Provider	NA	50% reduction in incidence; 17% reduction in child mortality	NA	NA	NA	Once a year: Malathion =$12; DDT =$9; Deltamethrin =$10; λ-cyhalothrin =$10; Twice a year: Malathion =$24; DDT =$17; Deltamethrin =$18; λ-cyhalothrin =$19	[1]
South Africa and Mozambique	SA =1997-1999; MZ =1999-2001	USD 2005	<5	Provider	SA =$3.27 and MZ =$3.90	5.5 child deaths averted per 1,000 child-years	NA	NA	SA =$4,357; MZ =$3,933	SA =$132; MZ =$119	[2]
SSA	Hypothetical 10 years program	Int 2000	<5	Provider	NA	50% reduction in incidence; 20% reduction in case fatality	Incidence: Afr-D= 1,436; Afr-E= 1,184. Mortality: Afr-D= 7; Afr-E = 8.	NA	NA	Afr-D =$32; Afr-E =$41	[3]
Kenya	1999-2000	~USD 2000	0-99	Provider	$0.88	RR=0.25 (0.24-0.27)	NA	$9	NA	NA	[4]
SSA (model)	NA	USD 1995	<1	Provider & Community	Once yearly ($5.76-10.18); Twice yearly ($11.53-20.36)	52% decrease in all cause mortality	1,500	NA	NA	Once yearly ($16-29); Twice yearly ($32-58)	[5]
Mozambique	0	USD 2000	2-15 (parasitaemia) & 0-99 (clinical case)	Provider	$4.82	11,857 malaria cases averted	NA	$29.43	0	NA	[6]
Model Area in Africa	1978	~USD 1975	0-99	Gross CER	$2	40% reduction in crude death rate (adult); 50% reduction in infant mortality	NA	NA	$250 (all); $600 (infants)	NA	[7]

Table 5 Summary of cost-effectiveness analyses for insecticide-treated bednet interventions conducted in sub-Saharan Africa.

Country	Year of Program	Currency	Age Group	Perspective	Cost PPY	Effect Size	Incidence per 1,000	CER / Infection	CER / Death	CER / DALY	Ref
Low and Middle Income Countries	Hypothetical Program	USD 2001	<5	Provider	NA	5.5 child deaths averted per 1,000 child-years	NA	NA	NA	Permetrhin =$17 Deltamethrin =$11	[1]
Eritrea (ER); Togo (TG); Malawi (MW); Senegal (SG); Tanzania (TZ);	ER=2001-2005; TG=2004; MW=1999-2005; SG=2000-2005; TZ=2002-2005	USD 2005	<5	Provider	Cost per ITN: ER=$3.98; TG=$3.32; MW=$3.36; SG=$8.05; TZ=$4.80	50% reduction in incidence; 20% reduction in case fatality	NA	NA	Including re-treatment: ER=$438; TG=$1,174; MW=$1,105; SG=$2,199; TZ=$788.	Including re-treatment: ER=$13; TG=$36; MW=$33; SG=$67; TZ=$24.	[2]
SSA	Hypothetical 10 years program	INT 2000	<5	Provider	NA	RR=0.25 (0.24-0.27)	Incidence: Afr-D=1,436; Afr-E=1,184. Mortality: Afr-D=7; Afr-E=8.	NA	NA	Afr-D =$29 Afr-E =$41	[3]
Kenya	1999-2000	~USD 2000	0-99	Provider	$2.02-2.34	19% decrease in all cause mortality; 46% decrease in malaria-related morbidity	NA	$29	NA	NA	[4]
SSA (model)	NA	USD 1995	<5	Provider & Community	Not mentioned	0.69 (ITN vs IRS)	1500	NA	NA	Bednet only: $19-85; Bednet and insecticide: $25-96	[5]

Country	Year of Program	Currency	Age Group	Perspective	Cost PPY	Effect Size	Incidence per 1,000	CER / Infection	CER / Death	CER / DALY	Ref
South Africa (KwaZulu-Natal)	1998-1999 (Comparison ITN vs IRS)	USD 1999	All	Provider	7.62	HR for infant mortality of 0.78	253 (174.5 /0.69)	16	$1,696	NA	[8]
Democratic Republic of the Congo	2005-2006 (Comparison ITN vs diagnosis / treatment, IPTp, / ANC services)	USD 2005	Pregnant women	Provider	Not mentioned	Reduction in all-cause mortality of 17% among U5	Used an infant mortality rate of 95/1,000	NA	$411.13	$17.22	[9]
Togo	2004-2005	USD 2004	<5	Provider	$5.95 (per LLIN distributed)	Reduction in incidence of 50% and 5.5 deaths averted per 1,000	1,209	4.4	$856	$22.1	[10]
United Republic of Tanzania	2004-2006	USD 2006	<5	Provider	$7.57 (per ITN delivered)	PE=27%	Incidence of malaria outpatient in U5 is 723/1,000	NA	$873	NA	[11]
United Republic of Tanzania	1996-2000	USD 2000	<5	Provider	$13.38 per treated-net year	NA (73 deaths averted)	Infant mortality =73; Child mortality =15	NA	$1,559	$57	[12]
Kenya	1997-1999	USD 1996	<5	Provider & Community	$1.40 ($1.90 per ITN)	Reduction in incidence of 46%; reduction in all cause mortality of 19%	NA	NA	$1,214	$49	[13]
Sub-Saharan Population	Not mentioned	USD 1995	1-119 months	Provider	3.79	Not mentioned	U5 =1,500; 5-10 =555	NA	NA	No rebound =$44	[14]

Country	Year of Program	Currency	Age Group	Perspective	Cost PPY	Effect Size	Incidence per 1,000	CER / Infection	CER / Death	CER / DALY	Ref
Guinea	Not mentioned	USD 1994	<5	Provider	3	50% reduction in incidence; 35% reduction in all-cause mortality	Mortality =14	NA	NA	$43	[15]
The Gambia	1990-1995	USD 1990	The cohort of children born in 1990 and followed for 5 years	Provider	NA	Estimated from 10 villages	1-6 months =200; 6-12 months =800; 1-5 years =1,000	NA	$711	NA	[16]
The Gambia	1991-1992	USD 1992	<10	Provider & Community	NA	U5 mortality reduced by 17%	440	NA	$471	$31.5	[17]
Ghana	1993-1994	USD 1994	<5	Provider & Community	$1.2 per child-year ($2.4 per bednet)	All-cause mortality reduced by 25% in U5.	NA	NA	$2,003	$73.5	[18]
West Africa	5 years of follow-up	~USD 1996	Cohort of new-born	Provider	Not mentioned	60% decrease in mortality in the 1-4 years old; 45% decrease in clinical episodes	Not mentioned	NA	NA	100% compliance = $18.88; 50% compliance = $38.04	[19]
The Gambia	1989-1990	USD 1990	1-4 years of age	Provider & Community	$5.65 per child-year	0	Not mentioned	NA	$187.53	$7.9	[20]

ANC = antenatal care; INT = international dollar; IPTp = intermittent treatment for malaria in pregnancy; ITN = insecticide treated nets; IRS = indoor residual spraying; LLIN = long-lasting insecticide-treated nets; NA = not applicable; U5 = under five years of age; USD = United States dollar.

REFERENCES:

1. Breman J, Mills A, Snow R, Jo-Ann Mulligan J, Lengeler C, Mendis K, Marchesini P, White N, Steketee R, Doumbo O: **Conquering Malaria.** In *Disease Control Priorities in Developing Countries.* 2nd Edition edition. Edited by Jamison DT, Breman JG, Measham AR, Alleyne G, Claeson M, Evans DB, Jha P, Mills A, Musgrove P. Washington, DC: World Bank; 2006

2. Yukich JO, Lengeler C, Tediosi F, Brown N, Mulligan JA, Chavasse D, Stevens W, Justino J, Conteh L, Maharaj R, et al: **Costs and consequences of large-scale vector control for malaria.** *Malar J* 2008, **7:**258.

3. Morel CM, Lauer JA, Evans DB: **Cost effectiveness analysis of strategies to combat malaria in developing countries.** *BMJ* 2005, **331:**1299.

4. Guyatt HL, Corlett SK, Robinson TP, Ochola SA, Snow RW: **Malaria prevention in highland Kenya: indoor residual house-spraying vs. insecticide-treated bednets.** *Trop Med Int Health* 2002, **7:**298-303.

5. Goodman CA, Coleman PG, Mills AJ: **Cost-effectiveness of malaria control in sub-Saharan Africa.** *Lancet* 1999, **354:**378-385.

6. Conteh L, Sicuri E, Manzi F, Hutton G, Obonyo B, Tediosi F, Biao P, Masika P, Matovu F, Otieno P, et al: **The cost-effectiveness of intermittent preventive treatment for malaria in infants in Sub-Saharan Africa.** *PLoS One* 2010, **5:**e10313.

7. Walsh JA, Warren KS: **Selective primary health care: an interim strategy for disease control in developing countries.** *N Engl J Med* 1979, **301:**967-974.

8. Goodman CA, Mnzava AE, Dlamini SS, Sharp BL, Mthembu DJ, Gumede JK: **Comparison of the cost and cost-effectiveness of insecticide-treated bednets and residual house-spraying in KwaZulu-Natal, South Africa.** *Trop Med Int Health* 2001, **6:**280-295.

9. Becker-Dreps SI, Biddle AK, Pettifor A, Musuamba G, Imbie DN, Meshnick S, Behets F: **Cost-effectiveness of adding bed net distribution for malaria prevention to antenatal services in Kinshasa, Democratic Republic of the Congo.** *Am J Trop Med Hyg* 2009, **81:**496-502.

10. Mueller DH, Wiseman V, Bakusa D, Morgah K, Daré A, Tchamdja P: **Cost-effectiveness analysis of insecticide-treated net distribution as part of the Togo Integrated Child Health Campaign.** *Malar J* 2008, **7:**73.

11. Mulligan JA, Yukich J, Hanson K: **Costs and effects of the Tanzanian national voucher scheme for insecticide-treated nets.** *Malar J* 2008, **7:**32.

12. Hanson K, Kikumbih N, Armstrong Schellenberg J, Mponda H, Nathan R, Lake S, Mills A, Tanner M, Lengeler C: **Cost-effectiveness of social marketing of insecticide-treated nets for malaria control in the United Republic of Tanzania.** *Bull World Health Organ* 2003, **81:**269-276.

13. Wiseman V, Hawley WA, ter Kuile FO, Phillips-Howard PA, Vulule JM, Nahlen BL, Mills AJ: **The cost-effectiveness of permethrin-treated bed nets in an area of intense malaria transmission in western Kenya.** *Am J Trop Med Hyg* 2003, **68:**161-167.

14. Coleman PG, Goodman CA, Mills A: **Rebound mortality and the cost-effectiveness of malaria control: potential impact of increased mortality in late childhood following the introduction of insecticide treated nets.** *Trop Med Int Health* 1999, **4:**175-186.

15. Jha P, Bangoura O, Ranson K: **The cost-effectiveness of forty health interventions in Guinea.** *Health Policy Plan* 1998, **13:**249-262.

16. Graves PM: **Comparison of the cost-effectiveness of vaccines and insecticide impregnation of mosquito nets for the prevention of malaria.** *Ann Trop Med Parasitol* 1998, **92:**399-410.

17. Aikins MK, Fox-Rushby J, D'Alessandro U, Langerock P, Cham K, New L, Bennett S, Greenwood B, Mills A: **The Gambian National Impregnated Bednet Programme: costs, consequences and net cost-effectiveness.** *Soc Sci Med* 1998, **46:**181-191.

18. Binka FN, Mensah OA, Mills A: **The cost-effectiveness of permethrin impregnated bednets in preventing child mortality in Kassena-Nankana district of Northern Ghana.** *Health Policy* 1997, **41:**229-239.

19. Evans DB, Azene G, Kirigia J: **Should governments subsidize the use of insecticide-impregnated mosquito nets in Africa? Implications of a cost-effectiveness analysis.** *Health Policy Plan* 1997, **12:**107-114.

20. Picard J, Aikins M, Alonso PL, Armstrong Schellenberg JR, Greenwood BM, Mills A: **A malaria control trial using insecticide-treated bed nets and targeted chemoprophylaxis in a rural area of The Gambia, west Africa. 8. Cost-effectiveness of bed net impregnation alone or combined with chemoprophylaxis in preventing mortality and morbidity from malaria in Gambian children.** *Trans R Soc Trop Med Hyg* 1993, **87 Suppl 2:**53-57.
